# Agreement between Arterial and Capillary pH, pCO_2_, and Lactate in Patients in the Emergency Department

**DOI:** 10.1155/2021/7820041

**Published:** 2021-07-06

**Authors:** Vincent Collot, Stefano Malinverni, Jabir Haltout, Eric Schweitzer, Pierre Mols, Magali Bartiaux

**Affiliations:** Emergency Department, Centre Hospitalier Universitaire Saint-Pierre, Université Libre de Bruxelles, Brussels, Belgium

## Abstract

**Background:**

Blood gas analysis (BGA) is a frequent painful procedure in emergency departments. The primary objective of the study was a quantitative analysis to assess the mean difference and 95% confidence interval of the difference between capillary and arterial BGA for pH, pCO_2_, and lactate. Secondary objective was to measure the sensitivity and specificity of capillary samples to detect altered pH, hypercarbia, and lactic acidosis. Adults admitted to the ED were screened for inclusion. We studied the agreement between the two methods for pH, pCO_2_, and lactate with Bland-Altman bias plot analysis and receiver operating characteristic curves.

**Results:**

One hundred ninety-seven paired analyses were included. Mean difference for pH between arterial and capillary BGA was 0.0095, and 95% limits of agreement (LOA) were −0.048 to 0.067. For pCO_2_, mean difference was −0.3 mmHg, and 95% LOA were −8.5 to 7.9 mmHg. Lactate mean difference was −0.93 mmol/L, and 95% LOA were −2.7 to 0.8 mmol/L. At a threshold of 7.34, capillary pH had 98% sensitivity and 97% specificity to detect acidemia; at 45.9 mmHg, capillary pCO_2_ had 89% sensitivity and 96% specificity to detect hypercarbia. At a threshold of 3.5 mmol/L, capillary lactate had 66% sensitivity to detect lactic acidosis.

**Conclusion:**

Capillary BGA cannot replace arterial BGA despite high concordance between the two methods for pH and pCO_2_ and moderate concordance for lactate. Capillary measures had good accuracy when used as a screening tool to detect altered pH and hypercarbia but insufficient sensitivity and specificity when screening for lactic acidosis.

## 1. Introduction

Arterial blood gas analysis (BGA) is universally used in emergency departments (ED) to assess for acid-base abnormalities, oxygenation, and ventilation status in patients with suspected severe illnesses. Arterial BGA involves an arterial puncture or the placement of an indwelling arterial catheter. Both techniques are painful [1], distressing for the patients and associated with complications in a significant proportion of cases, such as hematomas, arterial lesions, ischemia, nervous lesions, or infections [2, 3].

Alternatives to arterial BGA exist. Continuous pulse oximetry is ubiquitously used and is both accurate and precise when compared to SaO_2_. Venous BGA, with or without mathematical arterialization of the results, is both moderately accurate and highly precise in terms of prediction of arterial pH [4–8]. With a threshold of 45 mmHg, venous pCO_2_ can be used as a sensible screening test for hypercarbia in COPD [9]; nevertheless, venous pCO_2_ values show only moderate accuracy in predicting arterial pCO_2_ [4, 5, 7] and cannot be used to replace arterial pCO_2_ [8, 10]. The agreement between venous and arterial lactate is sufficient only to rule out hyperlactatemia in septic patients when this is absent in venous blood [11]. Altogether these results suggest that VBG might be used as a screening tool in emergency care when coupled with clinical reasoning [12] but better alternatives should be investigated.

Capillary blood for BGA is widely adopted for the assessment of the acid-base status and pCO_2_ in pediatrics worldwide [13–16]. It is associated with extremely rare complications [17], reduced discomfort [18], and an easier sampling technique requiring less qualified personnel. In adult population, capillary BGA is not as disseminated as in child population. Adult BGA acquired from arterialized samples, obtained by warming the skin or applying a vasodilator substance, showed a good agreement with arterial BGA in terms of pH and pCO_2_ [18–20]. Nevertheless, arterialization is time consuming, preventing its use beyond research purposes. A recent study from an ICU population showed that similar results could be obtained without prior arterialization of the capillary bed [21]. A meta-analysis showed encouraging results for capillary BGA accuracy, but given its heterogeneous population composed partially of healthy subjects, these results cannot be extrapolated to emergency care. Moreover, capillary BGA studies conducted in the ED reported only correlation coefficients without studying mean difference and 95% limits of agreement, answering incompletely on whether capillary BGA could replace arterial BGA [22].

The primary objective of the study was to assess the agreement between capillary BGA and the reference gold standard technique arterial BGA in terms of pH, pCO_2_, and lactate through the Bland-Altman agreement plot technique [23]. The secondary outcome of our study was to identify and describe the performance of capillary BGA to detect altered pH, hypercarbia, and lactic acidosis at different thresholds.

## 2. Materials and Methods

We conducted a prospective monocentric study on a convenience sample of patients admitted to the ED from December 2017 to April 2018. Adults presenting to the ED for whom the treating clinician deemed an arterial BGA necessary were screened for inclusion if not pregnant. Screening was performed according to study personnel availability and overall workload. We calculated a sample size of 200 patients based on a previous study [21] reporting a standard deviation of differences for pH of 0.0338. We based our sample calculation on the precision of the estimates and aimed for a standard error of the 95% limit of agreement of approximately ±0.01.

Simultaneous arterial and capillary samples were drawn for BGA. Arterial BGA were sampled by direct arterial radial or femoral puncture using a preheparinized syringe (safe-PICO aspirator®, 1.7 ml, Radiometer®). Capillary samples were obtained at patients' finger using a contact-activated lancet (BD Microtainer®, Becton Dickinson®) and collected through capillary tubes (safeCLINITUBES® 70 *μ*l, Radiometer®). Samples were analyzed with the same point of care BGA analyzer (ABL90 FLEX® Radiometer®). Analysis was not blinded, and clinical information, index test results, and reference standard results were available to the assessors of the reference standard.

The Shapiro–Wilk test was used to evaluate the normal distribution of the data.

The agreement between the two methods for pH, pCO_2_, and lactate was studied using Bland-Altman bias plot analysis. The upper and lower 95% limits of agreement were calculated as the mean difference ±1.96 standard deviation of the difference. Pearson's product-moment correlation coefficients were calculated to assess the strength and direction of association between values of pH, pCO_2_, and lactate obtained through capillary and arterial puncture.

The reported analytical error for pH following an internal quality control showed a standard deviation of 0.0016 for repeated measures at 7.17. The reported analytical error for pCO_2_ measurements was associated with a standard deviation of 0.228 when pCO_2_ was 65.9 mmHg. The reported analytical error for lactate was associated with a standard deviation of 0.52 when lactate was at 3.55 mmol/L.

We considered that a capillary measure could replace an arterial measure if the double of the standard deviation of the bias of pH, pCO_2_, or lactate would be inferior to the reference analytical error (bias + 1.65SD) of the laboratory for the parameter at study.

Sensitivity, specificity, positive and negative predictive values, and AUC were calculated for the ability of capillary samples to detect pH values outside normal ranges, hypercarbia, and hyperlactatemia. Cutoff values were determined using the Liu method which maximizes the product of the sensitivity and specificity.

A post-hoc subgroup analysis to identify the variables accounting for variability in diagnostic accuracy was performed.

Missing data were handled as missing, and no imputation was performed.

The study was approved by the Ethical Committee of Saint-Pierre (B076201630203), and informed consent was obtained from all participants.

## 3. Results

**Figure 1:**
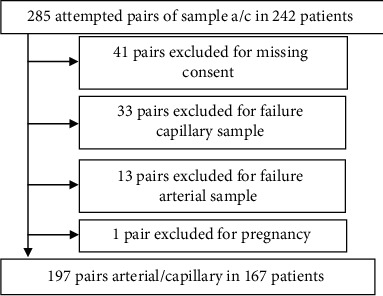
Inclusion flowchart showing the screening, exclusion, and analysis populations.

During the study period, 1298 BGA on 1170 patients were performed, and 242 patients underwent screening, generating 285 pairs of arterial and capillary BGA. 41 declined consent, and one subject was excluded as discovered pregnant after inclusion. In 13 cases, the arterial sample was inappropriate or unavailable: in three subjects, no arterial blood could be obtained, and in ten cases, there was a mixture of venous and arterial blood. In 33 cases, the capillary sample was inappropriate: in 15 cases, blood drawn in the capillary tube was insufficient; in six cases, capillary blood could not be obtained from the patient; in five cases, air bubbles were present in the tube; and in five subjects, unspecified problems hampered the analysis. After applying exclusion criteria, a total of 197 paired BGA, from 167 subjects, were included (Figure 1).

The mean age of subjects was 57.9 years (±16.3), and 64% were men. Baseline characteristics, hemodynamic variables, and clinical diagnoses are reported in Table 1.

Acidemia, defined as a pH below 7.35, was present in 24% of arterial samples while alkalemia, defined as a pH above 7.45, was present in 21%. Hypercarbia, defined as a pCO_2_ above 45 mmHg, was present in 33% of arterial samples, and elevated lactate, defined as lactate above 2 mmol/L, was found in 26%. The median delay between arterial and capillary sampling was one minute.

**Figure 2:**
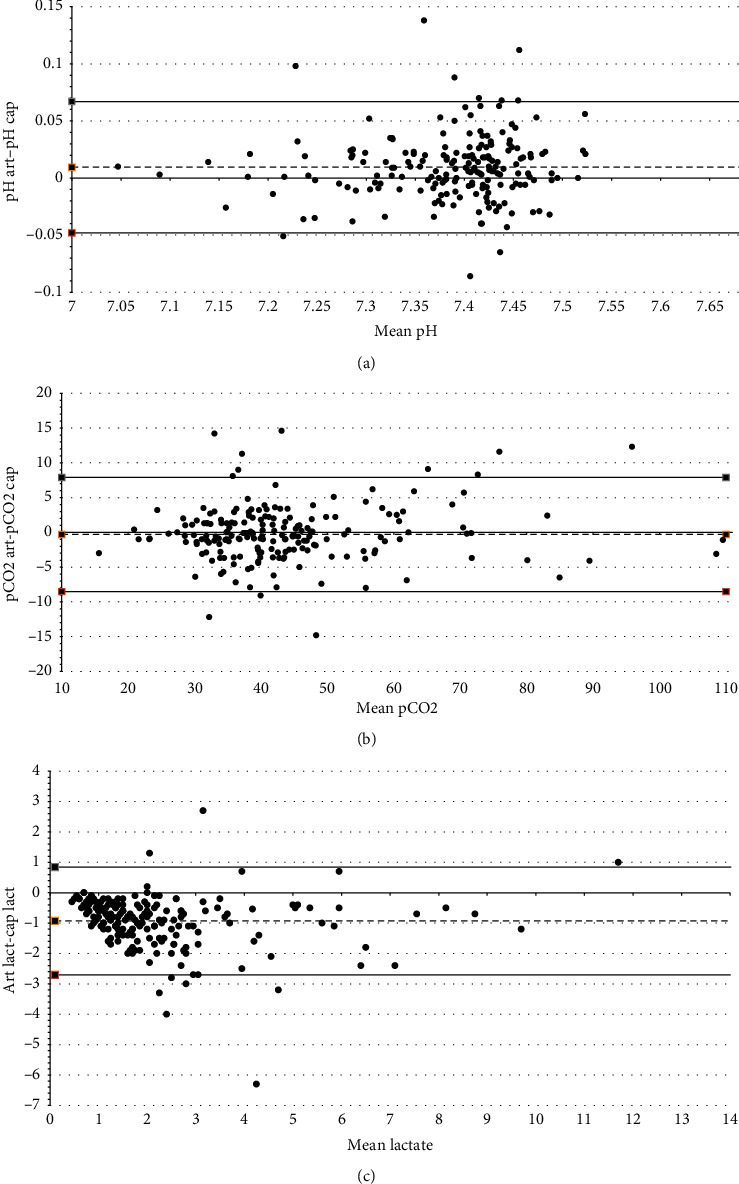
Bland-Altman agreement plots for paired measures of pH, pCO_2_, and lactate. (a) Bland-Altman arterial pH-capillary pH. (b) Bland-Altman arterial pCO_2_-capillary pCO_2_. (c) Bland-Altman arterial lact-capillary lact. (a) The bias was 0.01 (95% limits of agreement −0.048–0.067) for paired capillary and arterial measures of pH. (b) The bias was −0.3 (95% limits of agreement, −8.5–7.9 mmHg) for paired measures of pCO_2_. (c) The bias was −0.93 (95% limits of agreement −2.7–0.84 mmol/L) for paired measures of lactate.

The median pH was 7.41 (IQR 7.36–7.44). The mean pH difference (i.e., bias) (arterial-capillary) was 0.01 with 95% limits of agreement ranging from −0.048 to 0.067 (Figure 2). The concordance correlation coefficient between arterial and capillary pH was 0.943 (95% CI, 0.928–0.959).

The median pCO_2_ was 40.8 mmHg (IQR 34.7–47.3). The mean pCO_2_ bias (arterial-capillary) was −0.3 mmHg with 95% limits of agreement ranging from −8.5 to 7.9 mmHg (Figure 2). The concordance correlation coefficient between arterial and capillary pCO_2_ was 0.963 (95% CI, 0.952–0.974).

The median lactate was 1.2 mmol/L (IQR 0.8–2.1). The mean lactate bias (arterial-capillary) was −0.93 mmol/L with 95% limits of agreement going from −2.7 to 0.84 mmol/L (Figure 2). The concordance correlation coefficient between arterial and capillary lactate was 0.824 (95% CI, 0.783–0.865). The percentage error was 78.8%.

**Figure 3:**
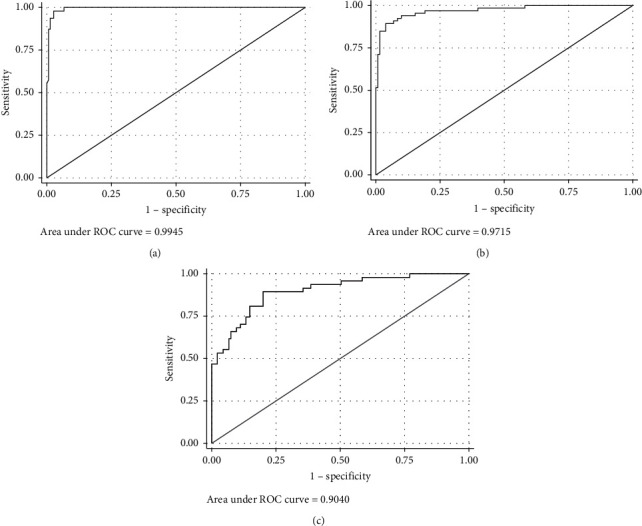
Receiver operating characteristic (ROC) curves for the diagnosis of acidosis, hypercapnia, and hyperlactatemia according to capillary samples. (a) Acidosis ROC curve (area under ROC curve = 0.9945). (b) Hypercapnia ROC curve (area under ROC curve = 0.9715). (c) Hyperlactatemia ROC curve (area under ROC curve = 0.9040).

The area under the receiver operating characteristic (ROC) curve to assess the ability of capillary BGA to detect arterial acidemia was 0.99 (95% CI, 0.99 to 1) (Figure 3).

The area under the curve for capillary samples was 0.99 (95% CI, 0.99 to 1) among the patients with acidosis, 0.97 (95% CI, 0.95–0.99) among the patients with hypercapnia, and 0.90 (95% CI 0.85–0.95) among the patients with hyperlactatemia.

A cutoff point of 7.34 capillary pH detected arterial acidosis with 98% sensitivity and 97% specificity (Table 2). The positive predictive value (PPV) of capillary pH below 7.34 to detect acidemia was 92% while the negative predictive value was 99%.

For each analysis, three different cutoff levels were analyzed presenting their results in terms of sensitivity, specificity, positive predictive value, and negative predictive value. The three cutoff levels were determined to, respectively, maximize the negative predictive value, the Liu coefficient, and the positive predictive value for capillary BGA in detecting altered pH, pCO_2_, or lactate.

The area under the ROC curve to assess the ability of capillary BGA to detect arterial hypercarbia was 0.97 (95% CI, 0.95–0.99) (Figure 3). At a cutoff of 45.9 mmHg, capillary pCO_2_ detected arterial hypercarbia with 89% sensitivity and 96% specificity. The PPV of capillary pCO_2_ above 45.9 mmHg to detect hypercarbia was 92%, while the negative predictive value was 94% (Table 2).

The area under the ROC curve constructed to assess the ability of capillary BGA to detect arterial hyperlactatemia was 0.90 (95% CI 0.85–0.95) (Figure 3). A threshold of 3.5 mmol/L for capillary lactate was associated with a sensitivity of 66% and a specificity of 93% for detecting hyperlactatemia. The PPV of capillary lactate above 3.5 mmol/L was 76%, while the negative predictive value was 89% (Table 2).

Sensitivity for detecting acidemia was 100% when using a pH cutoff value of 7.36 and was associated with a specificity of 93% and a PPV of 0.82 (Table 2).

The sensitivity for detecting hypercarbia was 100% when using a pCO_2_ cutoff value of 35.8 mmHg and was associated with a specificity of 42% and a PPV of 0.47.

For lactate, the sensitivity for detecting hyperlactatemia was 100% when using a cutoff value of 1.3 mmol/L and was associated with a specificity of 23% and a PPV of 0.31.

A subgroup analysis according to delays between samples, presence of hypotension, and suspected pathology did not yield any difference between subgroups (Table 3).

Sensitivity analysis was to assess agreement of capillary BGA and arterial BGA according to differences in sampling delays, hemodynamic state, and suspected admission diagnosis. No substantial heterogeneity was observed between groups.

Data supporting the study are available at https://doi.org/10.17632/dxy4dc86nc.1.

We observed no major complication associated with either technique, apart from pain during the arterial sampling.

## 4. Discussion

This study indicates that capillary blood sampling to assess pH and pCO_2_ has high accuracy and moderate precision when compared to the gold standard technique of arterial blood sampling. Accuracy and precision in terms of predicting arterial lactate from capillary values were lower.

The double of the standard deviation of the bias of pH, pCO_2_, and lactate was superior to the reference analytical error (bias + 1.65SD) of the laboratory for the parameter at study. Therefore, capillary sampling alone cannot replace arterial sampling for BGA in an ED.

Nevertheless, ROC curves analysis showed that capillary pH is highly predictive of arterial acidemia. Using a threshold of 7.36 for capillary pH yielded a sensitivity of 100% and a specificity of 93% in detecting acidemia with a negative predictive value of 100%. This means it could be used as a triage tool in subjects with suspected respiratory acidosis to select patients needing further assessment through arterial BGA, thus avoiding useless, painful, and potentially harmful arterial sampling in a substantial proportion of patients presenting in the ED for a respiratory condition. Within 59 patients presenting for an obstructive pulmonary condition, this strategy would have avoided 37 (62.7%) arterial BGA, assuming the aim of the analysis was to detect respiratory acidosis. Capillary BGA with the same threshold applied to 16 subjects with decompensated diabetes suspected of diabetic ketoacidosis would have avoided 15 (93.8%) arterial BGA, assuming the aim of the analysis was to detect arterial acidemia only.

A recent meta-analysis of studies comparing venous BGA to arterial BGA showed a mean difference for pH between the two techniques of 0.033 and limits of agreement ranging from −0.023 to 0.090 [8], in line with what other studies focused on the ED [4, 24, 25] have reported. Our study shows a lower mean difference of 0.01 and similar limits of agreement going from −0.048 to 0.067 when comparing arterial BGA to capillary BGA, suggesting that the latter might be a better alternative than venous BGA. When comparing pCO_2_ values from venous BGA to values obtained from arterial BGA, a recent review article showed a mean difference for pCO_2_ of 5.7 mmHg with limits of agreement falling outside ±10 mmHg in most of the cases [24]. Our study shows a mean difference of −0.3 mmHg and limits of agreement going from −8.5 to 7.9 mmHg when comparing arterial to capillary pCO_2_ suggesting that capillary BGA might be a more accurate alternative than venous BGA.

Our study shows an accurate detection of arterial hypercarbia through capillary BGA with an AUC of 0.97. When using a threshold of 45.9 mmHg, capillary BGA had a good positive and negative predictive value for detecting hypercarbia (Table 2).

According to study results, capillary lactate levels are only moderately accurate and precise in their prediction of arterial lactate levels. Capillary lactate levels tend to be systematically higher than arterial levels and with a high percentage error. Capillary lactate measurement does not have an adequate agreement with arterial BGA for clinical use.

These results suggest that capillary BGA can reliably be used as a screening tool for acidemia and hypercarbia in patients suspected for alteration of both pH and pCO_2_ in the ED avoiding a substantial proportion of arterial BGA, the pain associated with the arterial procedure, and accelerating the care process with a less skill-intensive procedure. These results urgently question some deep-rooted routine procedures of widespread arterial BGA utilization since less painful, safe, and precise alternatives exist for the initial screening of patients in the ED.

Cost associated with capillary sampling may be context-specific. In our context, capillary sampling and analysis costs were only slightly higher than those for arterial BGA. As no additional equipment is needed except for the capillary tubes, the gain associated with capillary BGA in terms of workload and workforce cost should encourage their implementation in ED.

Our study has strengths and limitations. One limitation is its monocentric design as it limits the ability to generalize our results to other ED and other settings. A second limitation is having used an ideal convenience sample of 200 patients. As inclusion was erratic, and according to workload, this might have introduced a bias in recruitment. The main strengths of our study are its pragmatic design with wide study population in terms of size and variety of pathologies together with the rigorous reporting of results beyond correlation analysis. Further studies should replicate our findings in different settings and test the implementation of capillary BGA for screening purposes.

## 5. Conclusions

To conclude, capillary samples for BGA have a high concordance for pH and pCO_2_ and moderate concordance for lactate when compared to arterial sampling. As its precision is insufficient, capillary sampling cannot replace arterial sampling. Capillary BGA has good sensitivity and specificity when used as a screening tool to detect altered pH and hypercarbia but insufficient sensitivity and specificity when screening for lactic acidosis.

## Figures and Tables

**Table 1:** Demographic and clinical characteristics of patients at study inclusion.

Number of observations	197
Age, mean (SD), *y*	57.9 (16.3)
Male, no. (%)	126 (63.9)
BMI, median (IQR)	24.7 (20.8–28.5)

Suspected pathology at sampling, no. (%)	
COPD	52 (26.4)
Asthma	7 (3.6)
Pneumonia	16 (8.2)
Pulmonary embolism	4 (2)
Other respiratory failure	22 (11.2)
Carbon monoxide poisoning	3 (1.5)
Diabetic ketoacidosis	16 (8.1)
Epilepsia	12 (6.1)
Sepsis	12 (6.1)
Shock	6 (3.1)
ROSC	3 (1.5)
Cardiac failure	15 (7.6)
CVA	1 (0.5)
Other	28 (14.2)

Capillary blood sampling attempts, no. (%)	
One attempt	170 (92.9)
Two attempts	12 (6.6)
Three or more attempts	1 (0.5)

Arterial blood sampling attempts, no. (%)	
One attempt	165 (90.7)
Two attempts	13 (7.1)
Three or more attempts	4 (2.2)

Mean arterial pressure (SD) (mmHg)	96.0 (21.1)
Delay arterial-capillary (IQR) (min)	1 (1–2)

BMI, body mass index; COPD, chronic obstructive pulmonary disease; CVA, cardiovascular accident; IQR, interquartile range; ROSC, return of spontaneous circulation SD, standard deviation.

**Table 2:** Cutoff levels, sensitivity, and specificity for capillary BGA in detecting altered pH, pCO_2_, or lactate.

Patients, no. (%)									
Value	Cutoff level	Sensitivity (%) (95% CI)	Specificity (%) (95% CI)	True positive	False negative	False positive	True negative	PPV (%) (95% CI)	NPV (%) (95% CI)
Arterial pH (acidosis)	7.361	100 (92.4–100)	93.3 (88.1–96.8)	47 (23.9)	0 (0)	10 (5.1)	140 (71.1)	82.5 (72.1–89.5)	100
	7.34	97.9 (88.7–100)	97.3 (93.3–99.3)	46 (23.7)	1 (0.5)	4 (2.0)	146 (74.5)	92 (81.4–96.8)	99.3 (95.5–99.9)
	7.288	53.1 (38.1–67.9)	100 (97.6–100)	25 (12.7)	22 (11.2)	0 (0)	150 (76.6)	100	87.2 (83.4–90.2)

Arterial pH (alkalosis)	7.4	100 (91.4–100)	62.2 (54.1–69.8)	41 (20.8)	0 (0)	59 (30)	97 (49.2)	41.0 (36.2–45.9)	100
	7.447	65.9 (49.4–79.9)	95.5 (91.0–98.2)	27 (13.1)	14 (7.1)	7 (3.5)	149 (75.6)	79.4 (64.4–89.2)	91.4 (87.4–94.2)
	7.471	39.0 (24.2–55.5)	100 (97.7–100)	16 (8.1)	25 (12.7)	0 (0)	156 (79.2)	100	86.2 (83.0–88.9)

pCO_2_ (mmHg) (hypercapnia)	35.8	100 (94.6–100)	42.0 (33.4–50.9)	66 (33.5)	0 (0)	76 (38.6)	55 (27.9)	46.5 (42.9–50.1)	100
	45.9	89.4 (79.4–95.6)	96.2 (91.3–98.8)	59 (29.9)	7 (3.6)	5 (2.5)	126 (63)	92.2 (83.3–96.6)	94.7 (89.9–97.3)
	56.5	51.5 (38.9–64.0)	100 (97.2–100)	34 (17.3)	32 (16.2)	0 (0)	131 (66.5)	100	80.4 (76.2–84.0)

Lactate (mmol/L) (hyperlactatemia)	1.3	100 (92.5–100)	23.0 (16.2–31.0)	47 (25.8)	0 (0)	104 (57.1)	31 (17.3)	31.1 (29.2–33.1)	100
	3.5	66.0 (50.7–79.1)	92.6 (86.8–96.4)	31 (17.3)	16 (8.8)	10 (5.5)	125 (68.7)	75.6 (62.3–85.4)	88.7 (84.0–92.1)
	4.4	46.8 (32.1–61.9)	100 (97.3–100)	22 (12.1)	25 (13.7)	0 (0)	135 (74.2)	100	84.4 (80.5–87.6)

PPV, positive predictive value; NPV, negative predictive value.

**Table 3:** Subgroup analysis according to delays in sampling and suspected diagnosis.

	pH			pCO_2_			Lactate		
	Bias	95% LOA		Bias	95% LOA		Bias	95% LOA	
Delay ≤ 1 min	0.009	−0.047	0.065	−0.24	−8.11	7.63	−0.88	−2.40	0.64
Delay = 2 min	0.011	−0.056	0.078	−0.38	−10.86	10.09	−0.94	−2.64	0.76
Delay > 2 min	0.012	−0.044	0.068	−0.82	−7.96	6.14	−1.2	−3.30	0.9
MAP ≥ 65 mmHg	0.010	−0.048	0.069	−0.43	−8.8	7.94	−0.91	−2.51	0.7
MAP ≤ 65 mmHg	0.002	−0.058	0.054	1.76	−6.88	10.39	−1.22	−2.48	0.03
Obstructive pulmonary disease	0.003	−0.052	0.059	0.15	−9.32	9.61	−0.85	2.00	0.3
Other respiratory failures	0.012	−0.059	0.083	−0.33	−10.25	9.59	−1.19	−3.01	0.62
Metabolic disturbances	0.014	−0.032	0.060	−1.21	−6.42	4.00	−0.89	−2.05	0.29
Cardiac failure	0.005	−0.042	0.053	−0.09	−8.49	8.3	−0.64	−2.43	1.14
Other	0.015	−0.047	0.078	−0.51	−7.88	6.87	−0.98	−2.89	0.93

BGA, blood gas analysis; LOA, limits of agreement; MAP, mean arterial pressure.

## Data Availability

The dataset supporting the conclusions of this article is available in the figshare repository, http://doi.org/10.17632/dxy4dc86nc.1.

## Supplementary Materials

Supplementary Materials Supplemental Digital Content Table 1: STARD-2015-Checklist EMI capillary.
